# Evaluation of the Salivary Matrix Metalloproteinase-9 in Women With Polycystic Ovaries Syndrome and Gingival Inflammation: A Case-Control Study

**DOI:** 10.7759/cureus.34458

**Published:** 2023-01-31

**Authors:** Avideh Maboudi, Fatemeh Nasiri Amiri, Sara Shafizade, Durdi Qujeq, Reza-Ali Mohammadpour, Amirhosain Moaddabi, Mania Amiri, Sadra Yosefnia-Pasha, Samih A Odhaib

**Affiliations:** 1 Department of Periodontology, Dental Research Center, Mazandaran University of Medical Sciences, Sari, IRN; 2 Department of Obstetrics and Gynecology, Infertility and Health Reproductive Research Center, Babol University of Medical Sciences, Babol, IRN; 3 Department of Dentistry, Mazandaran University of Medical Sciences, Sari, IRN; 4 Department of Clinical Biochemistry, Babol University of Medical Sciences, Babol, IRN; 5 Department of Statistic and Epidemiology, Mazandaran University of Medical Sciences, Sari, IRN; 6 Department of Oral and Maxillofacial Surgery, Mazandaran University of Medical Sciences, Sari, IRN; 7 Department of Obstetrics and Gynecology, Obstetricians, Infertility and Health Reproductive Research Center, Health Research Institute, Babol University of Medical Sciences, Babol, IRN; 8 Department of Dentistry, Babol University of Medical Sciences, Babol, IRN; 9 Department of Adult Endocrinology, Thi Qar Specialized Diabetes, Endocrine and Metabolism Center (TDEMC) Thi Qar Health Directorate, Thi Qar, IRQ

**Keywords:** oral health, polycystic ovary syndrome (pcos), matrix metalloproteinase-9, periodontal disease, gingivitis

## Abstract

Background

Polycystic ovary syndrome (PCOS) is an endocrine disease of women of reproductive age that impacts their oral and systemic well-being. This study aimed to compare the gingival inflammation indices and matrix metalloproteinase-9 (MMP-9) of non-obese women with PCOS.

Materials and methods

This is a case-control study in which 78 women were referred to the Babol Clinic Hospital in Northern Iran between 2018 and 2019. They were divided into three groups: 26 women with PCOS and gingivitis, 26 women with PCOS with no gingivitis, and 26 women with no PCOS and no gingivitis as a control group. After recording the anthropometric and demographic variables, fasting saliva samples were taken from all participants before any periodontal intervention. These samples were transferred to Babol Molecular Cell Research Center under highly guaranteed cold-chain conditions to measure the serum levels of MMP-9. Periodontal status was evaluated for Gingival Index (GI), Plaque Index (PI), and Bleeding on Probing (BOP). Analysis of variance was used to compare the mean results for these indices. The significance level was considered when p ≤ 0.05.

Results

All the gingival indices were significantly higher for women with PCOS with gingivitis compared to the results for women from the other two groups. Similarly, women with PCOS showed high salivary MMP-9 levels but were within the normal reference ranges.

Conclusion

The gingival indices (GI, PI, and BOP) and salivary MMP-9 are higher in women with PCOS, regardless of the gingival status.

## Introduction

Polycystic ovary syndrome (PCOS) is one of the most common women endocrinopathies [1], which is caused by multifactorial etiology based on the interaction between genetic, environmental, and hormonal factors [2,3]. The estimated prevalence of PCOS in women of reproductive age from Iran ranges from 5.8% to 19.5%, according to the different diagnostic criteria of PCOS [4,5].

Being a systemic disorder, there are several evidences of the relationship between gingivitis and systemic disorders, including PCOS, diabetes, and cardiovascular disease [6]. Since both gingivitis and PCOS are associated with systemic inflammation and insulin resistance, these two disorders might share a common pathophysiological pathway [7].

Studies suggest a potential link between this periodontal disease and PCOS through activation of different proinflammatory incidents like activation of different reactive oxygen species, myeloperoxidase, oxidative stress, inflammatory cytokines (such as IL-6 and TNF-α), high-sensitivity C-reactive protein (hs-CRP), adhesion molecules, and blood lymphocytes and monocytes [8-11]. However, some studies showed contradictory evidence [12].

Matrix metalloproteinase-9 (MMP-9) is a 92-kilodalton protein with protease activity whose main substrate is extracellular matrix and basement membrane tissue connections. It is the only family member that can attach and digest collagen as the most important component of the basement membrane due to its 3-fibronectin structure [13]. MMPs are found in a variety of environments. They have a very important role in migrating lymphoid and myeloid cells' physiological rearrangement of tissues, including organogenesis, normal growth, embryonic growth, angiogenesis, and ovulation [14]. This study aims to evaluate the possible linkage between different indices of gingival health, including the salivary MMP-9, to the polycystic ovary status in women with PCOS from Babol Northern Iran.

## Materials and methods

The present study is a case-control study conducted on women with PCOS who were referred to the Babol Clinic Hospital-Northern Iran between 2018 and 2019. The inclusion criteria for the case group were women aged 18 to 40 diagnosed with PCOS based on the Rotterdam diagnostic criteria [1]. The inclusion criteria for the women in the control group were those referred to the clinic for gynecological causes other than PCOS, with a normal menstrual cycle, without any clinical or biochemical indicators of hyperandrogenism, and no polycystic ovarian changes in their pelvic ultrasonography.

Exclusion criteria included any recent or current smoking history, women with diabetes mellitus, any history of malignancy, congenital adrenal hyperplasia, Cushing's syndrome, and androgen-secreting tumors, current smoking, intake of oral or injectable contraceptives within the last three months, history of antiepileptics or any drugs which are known to affect the gingiva by any level. Women having < 20 teeth and women with periodontitis were excluded.

Groups of body mass index (BMI) in kg/m^2^ were considered. If BMI is < 18.5, it falls within the underweight range. If BMI is 18.5 to <25, it falls within the healthy weight range. If BMI is 25.0 to <30, it falls within the overweight range. If BMI is 30.0 or higher, it falls within the obesity range [15].

A specialist gynecologist (assistant professor) examined all enrolled women. The women were diagnosed with PCOS according to the criteria above and then referred to the board-certified dentist after gaining the proper consent to be enrolled in the study. The latter examined the enrolled women for their periodontal state as either periodontally healthy or with gingivitis. The dentist in charge was blind to the PCOS status of women. If according to the depth of probing and clinical attachment loss, had periodontitis, it would be excluded from the study.

The gingival examination includes the Gingival Index (GI) (Silness and Löe), Plaque Index (PI) (Löe Index), Bleeding on Probing (BOP), and the number of existing teeth. To calculate the sample size, using the ratio of means method, the number of samples in each group (PCOS with gingivitis, PCOS with periodontally and systemically healthy control group) was calculated to be 26 persons with a 95% confidence level and 90% power. The enrolled women were divided into three groups: (Group 1) included women with PCOS and gingivitis, (Group 2) included women with PCOS with no gingivitis, and (Group 3) included women without PCOS or gingivitis.

Before the study's beginning, the measurement's reliability was determined by calculating the correlation coefficient for GI, PI, and BOP measurements. For this purpose, each index was measured twice in five women with gingivitis within one week. The results of these two examinations in terms of measurement criteria using correlation coefficient for quantitative indicators and kappa coefficient for quality index (BOP) between 80% and 85% was acquired, which indicated that the measurement had good reliability.

Details of periodontal examination

After receiving informed consent from the enrolled women, the periodontal examination was performed in the case and the control group using the mirror and the periodontal probe (William's probe) made by Medisporex-Pakistan. For this purpose, the Ramfjord teeth, including maxillary right first molar (Tooth 16), maxillary left central incisor (Tooth 11), maxillary left first bicuspid (Tooth 24), mandibular left first molar (Tooth 36), mandibular right central incisor (Tooth 41) and mandibular right first bicuspid (Tooth 44) were evaluated for GI, PI, and BOP. The adjacent tooth was evaluated as recommended if any Ramfjord teeth were pulled out in the past [16].

The periodontal disease diagnosis was based on inflammation in the gum tissue and the periodontium. In the case of gingivitis, the gums become erythematous, edematous, and easily bled during any intervention, such as probing with a periodontal probe. While healthy gum is coral pink, firm in consistency, does not bleed easily during probing, had normal gingival sulcus depth, and normal bone height according to the latest classification scheme for periodontal diseases and conditions [17]. The participants' periodontal state was recorded as either healthy or with mild, moderate, or severe gingivitis.

Details of MMP-9 evaluation

After the periodontal inspection to ensure no blood contamination, fasting saliva samples were taken from all participants in the morning before any periodontal intervention. The samples were transferred to sterilized micro-tubes and then sent to the Cellular and Molecular Biology Research Center at Babol University of Medical Sciences under the proper cold-chain conditions to measure the saliva levels of human MMP-9 using the quantitative single-wash 90 minutes Sandwich Enzyme-Linked Immunosorbent Assay (ELISA) kits. The sensitivity of this test was 22.17 pg/mL, with a range of (105.47-675 pg/mL).

Statistical analyses

The data were analyzed using IBM Software Statistical Packages for Social Sciences (SPSS) Version 26.0 (IBM Corporation, Armonk, NY). Analysis of Variance (ANOVA) was used to compare the quantitative variables for the overall groups and for in-between group comparison. We could not have a normal distribution for any test parameter, even with the log transformation of the parameters. A significance level (p≤0.05) was chosen to indicate a significant association.

Ethical principles of the research

This study was approved by the Ethics Committee of Mazandaran University of Medical Sciences on October 1, 2018, under the ethical approval code (IR.MAZUMS.REC.1397.2934). The participants were assured that their participation was voluntary, their data would remain confidential, and their autonomy would be respected. Written informed consent was obtained from each one of them.

## Results

**Figure 1:**
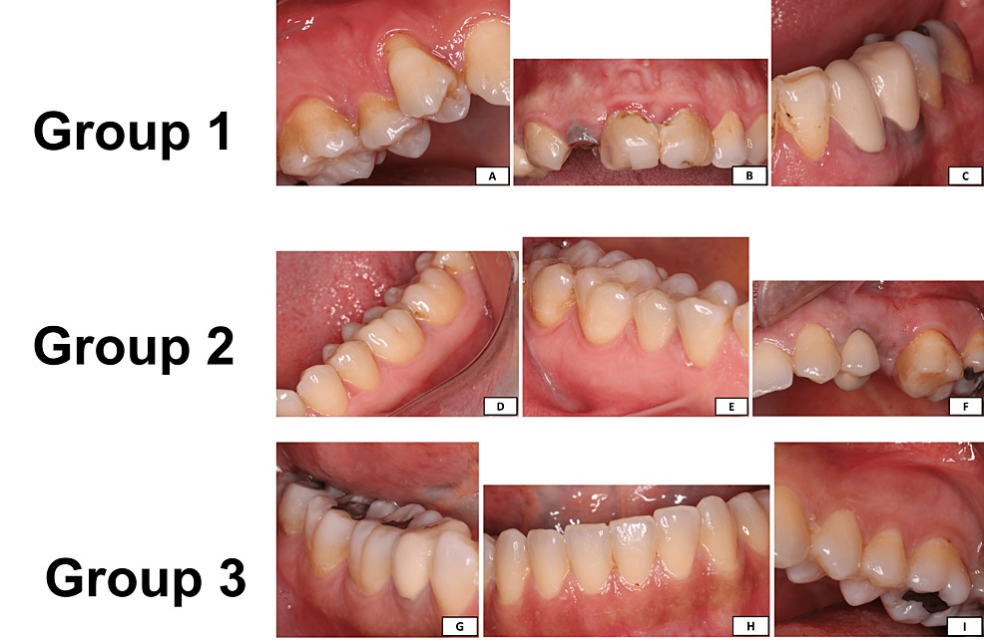
The different periodontal features the in the enrolled women. Group 1 images (A-C) are images from women with PCOS and gingivitis. Group 2 images (D-F) are images from women with PCOS and no gingivitis. Group 3 images (G-I) are images from women with neither PCOS nor gingivitis. (A) Right posterior maxillary gingival features of a patient with gingivitis and PCOS. (B) Anterior maxillary gingival features of a patient with gingivitis and PCOS. (C) Left lateral incisor to the second molar of mandibular gingival features of a patient with gingivitis and PCOS. (D) Left posterior mandibular gingival features of a patient with PCOS and normal periodontium. (E) Right posterior mandibular gingival features of a patient with normal periodontium and PCOS. (F) Left posterior maxillary gingival features of a patient with normal gingival and PCOS. (G) Right posterior mandibular gingival features of a patient with normal periodontium and without PCOS. (H) Anterior mandibular gingival features of a patient with normal periodontium and without PCOS. (I) Left posterior maxillary gingival features of a patient with normal periodontium and without PCOS. Abbreviations: PCOS, Polycystic Ovary Syndrome

A total of 78 women were enrolled in the study, all were never smokers, with 26 women in each group. The ranges of the age, BMI, and teeth count of the enrolled women were (16-40 years old), (18.70-36.60 kg/m^2^), and (25-32 teeth), respectively. Although the results of the parameters mentioned above were nearly similar and comparable between the three groups, they were significantly higher in women with PCOS and gingivitis. The periodontal indices for lower and upper jaws are significantly higher for women with PCOS and gingivitis than women from the other two groups. Figures 1A-1I represent different periodontal images for the enrolled women from the three groups.

**Table 1:** Evaluation of distribution of different parameters in the three groups of the study All parameters are illustrated as (Mean ± Standard Deviation) Abbreviations: MMP, Matrix Metalloproteinase; PCOS, Polycystic Ovary Syndrome

Parameters		PCOS with Gingivitis (n=26)	PCOS without Gingivitis (n=26)	Control (n=26)	P-value
Age years		27.19 ± 5.076	26.96 ± 5.66	23.42 ± 1.21	0.004
Body Mass Index kg/m^2^		27.89 ± 4.96	25.66 ± 4.14	22.34 ± 1.72	< 0.005
Teeth Count		28 ± 1.29	29.88 ± 1.53	29.92 ± 1.13	< 0.005
Gingival Index	Upper	96.12 ± 23.10	22.81 ± 10.49	24.73 ± 6.50	< 0.005
	Lower	94.77 ± 18.75	23.19 ± 9.15	20.62 ± 5.28	< 0.005
Plaque Index	Upper	84.73 ± 32.91	42.27 ± 19.85	20.85 ± 5.84	< 0.005
	Lower	87.81 ± 34.72	43.69 ± 21.43	19.81 ± 7.65	< 0.005
Bleeding on Probing	Upper	28.89 ± 12.02	8.00 ± 4.00	5.85 ± 3.00	< 0.005
	Lower	28.50 ± 9.29	7.65 ± 3.09	5.50 ± 2.61	< 0.005

**Figure 2:**
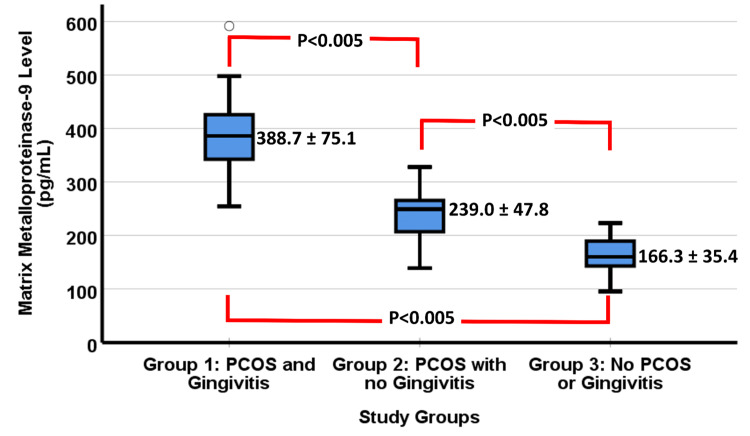
Boxplot of the results of MMP-9 in women from the three groups of the study. The overall p-value by ANOVA was < 0.005. The mean of the MMP-9 is illustrated on the left of each boxplot. The significance level of in-between groups comparison was measured. The single faint circle represents a single outlier result in one woman with PCOS and gingivitis.

Although all the results of the MMP-9 in all groups are within the normal reference ranges, yet women with PCOS and gingivitis had significantly higher ranges than other groups. Figure 2 illustrates the results of the MMP-9 for women in all groups. Women with PCOS and gingivitis had significantly higher MMP-9 than all groups. This result was evident by the overall mean comparison and the in-between group comparison using ANOVA. Women with PCOS but no gingivitis had significantly higher MMP-9 than women who had neither PCOS nor gingivitis.

## Discussion

Blood and saliva are valid sources for the estimation of many biomarkers like MMP-9, and there is evidence that serum and salivary MMP-9 show significantly increased levels in women with PCOS and gingivitis, which may indicate an exaggerated effect of the gingival inflammatory process in PCOS [12,18]. For the sake of convenience, we chose salivary samples in this study.

Relatively higher levels of MMP-9, the proinflammatory markers in saliva, may be a manifestation of low-grade systemic inflammation associated with PCOS [19]. Per the present study, systematic reviews by Márquez-Arrico et al. and Kellesarians et al. have confirmed that a positive association between periodontal pathologies and PCOS, particularly gingivitis and chronic periodontitis [19,20]. Likewise, Wendland et al. (2021) concluded that young women with PCOS and good oral health maintenance do not vary from healthy controls concerning gingival indices [21].

In the study of Dursun et al., clinical periodontal parameters similar to ours were compared in 25 women with PCOS and 27 healthy control women. These indices in tested women with PCOS were higher than in the women control group. They showed the volume of the gingival crevicular fluid, as well as the amount of nitric oxide (NO) and myeloperoxidase (MPO) in the gingival sulcus, was higher among the women with PCOS. Their findings suggested that the local/periodontal oxidant status in women with PCOS was damaged, and their susceptibility to periodontal diseases increased considerably [22].

In the present study, although all the readings of MMP-9 fall within the normal reference range, they were higher in women with PCOS regardless of their gingival status compared to women from the control group. The possible linkage between MMP-9 and PCOS pathogenesis is that the rate of growth and regeneration of ovarian follicles is modified by the balance between MMPs in the extracellular environment [23], which may explain the higher levels seen in women with PCOS compared to women in the control group in some studies [24-26]. Other studies provided contradictory results [27-29].

The small sample size, being a single-center cross-sectional observational study, limited the generalizability of the results, and fail to ensure causality. Additionally, this study did not convey a comparable evaluation of other proinflammatory and inflammatory indicators of PCOS and similar conditions.

## Conclusions

The indicators of gingival inflammation (GI, PI, and BOP) were higher in women with PCOS compared to women with PCOS with healthy gingiva, women in the control group. Although salivary MMP-9 was significantly higher for women with PCOS regardless of the gingival status compared to women in the other two groups, the levels were within the normal reference ranges, which will ameliorate the effect of such finding on the possible linkage to PCOS pathogenesis. Still, this association should be emphasized in the management plan of women with PCOS by regular referral for maintaining optimal dental and gingival care. Gingival and periodontal health status could mirror the ovarian status in women with PCOS to an extent, but this needs to be implicated in large-scale longitudinal studies in multicenter settings are recommended.
